# Locust cGAS-like Receptors Recognize Derivatives of a Gypsy Retrotransposon to Synergize with RNAi Against Viral Invasion

**DOI:** 10.3390/insects17060539

**Published:** 2026-05-22

**Authors:** Yi-Lan Li, Ma-Cheng Zhang, Shuo Yang, Peng Wang, Yao Xu, He-Ying Qian

**Affiliations:** Jiangsu Key Laboratory of Sericultural and Animal Biotechnology, School of Biotechnology, Jiangsu University of Science and Technology, Zhenjiang 212100, China; yilanli777@163.com (Y.-L.L.); 19505565016@163.com (M.-C.Z.); 18052146953@163.com (S.Y.); 19816476313@163.com (P.W.)

**Keywords:** *Locusta migratoria*, Gypsy retrotransposon, viral DNA, cGAS-like receptors, antiviral immunity

## Abstract

Transposable elements (TEs) are increasingly recognized as modulators of innate immunity, yet their antiviral functions remain poorly understood outside mammals and dipterans. Here, we identify a long terminal repeat retrotransposon, *LmGypsy*, as a key regulator of antiviral defense in *Locusta migratoria*. The infection of *Acrididae reovirus* (ARV) induces rapid upregulation of *LmGypsy*, and its inhibition compromises antiviral resistance. Mechanistically, *LmGypsy* promotes viral-derived DNA (vDNA) production, which drives *Dicer-2*-dependent biogenesis of virus-derived small interfering RNAs (vsiRNAs) to enhance RNA interference-mediated viral clearance. Notably, vDNA persists throughout infection, suggesting a role in sustaining antiviral responses. In parallel, LmGypsy activity is positively associated with induction of cyclic GMP-AMP synthase (cGAS)-like receptors (*LmcGAS1/2/4*) and their downstream effector Stimulator of Interferon Genes (*STING*). Together, these findings support a dual-layer antiviral strategy and indicate that TE-mediated immunity represents a widespread antiviral mechanism across taxa.

## Simple Summary

Once viewed as genomic parasites, transposable elements (TEs) can also defend hosts against infection. In *Locusta migratoria*, we find that a Gypsy retrotransposon, *LmGypsy*, is surprisingly beneficial during *Acrididae reovirus* (ARV) infection. Upon viral challenge, *LmGypsy* becomes activated and initiates a two-pronged immune response. It promotes the production of ARV-derived DNA to amplify RNA interference-mediated antiviral immunity and activates cGAS-like immune signaling. Loss of *LmGypsy* significantly increases host susceptibility to ARV infection. These findings identify TE-mediated immunity as a potentially conserved antiviral strategy across animals.

## 1. Introduction

Transposable elements (TEs), once regarded as genomic parasites or “junk DNA”, are now recognized as major drivers of genome evolution and functional innovation [1,2,3,4]. Across diverse eukaryotes, TEs have been a source of novel genes with critical host functions, contributing to phenotypic plasticity, environmental adaptation, and stress responses [5,6,7,8]. For example, in *Drosophila melanogaster*, the transcriptional regulation of stress-response genes is modulated by multiple families of TEs [9]. Beyond stress responses, TEs have also been implicated in higher-order biological processes, including synaptic plasticity, cognition, and tissue development and morphogenesis [10,11]. In particular, accumulating evidence indicates that TEs can participate in host defense, suggesting that these mobile genetic elements are not merely genomic liabilities but can be repurposed as functional components of immune systems [12,13].

The interplay between TEs and innate immunity is especially evident during viral infection [14,15]. While host genomes typically suppress TE activity to maintain genomic stability, infection can trigger transient TE derepression [16,17]. This process generates nucleic acid intermediates that resemble pathogen-associated molecular patterns (PAMPs), thereby amplifying immune signaling [15,16,18]. In mammals, endogenous retroelements such as long interspersed nuclear element-1 (*LINE-1*) produce cytosolic DNA species that activate the cGAS–STING (cyclic GMP-AMP synthase–stimulator of interferon genes) pathway and enhance antiviral responses [19,20]. Similarly, in insects, retrotransposon-encoded reverse transcriptases can convert viral RNA into viral-derived DNA (vDNA), which promotes RNA interference (RNAi)–mediated antiviral immunity [21,22,23,24]. These findings support a general model in which TEs reactivation acts as an intrinsic immune amplifier across taxa.

Despite these advances, the molecular principles linking TE activation to antiviral immunity remain incompletely understood [25]. Specifically, it is unclear how TE-derived nucleic acids are integrated into multiple immune pathways and whether such mechanisms are conserved beyond well-studied model systems [14]. Most current evidence derives from Diptera or vertebrate systems [3,15], leaving a substantial gap in our understanding of TE-mediated immunity in other insect orders. Moreover, the extent to which TE activity coordinates distinct antiviral pathways, such as RNAi and nucleic acid sensing, has not been systematically investigated [26].

The migratory locust (*Locusta migratoria*) provides a unique system to address these questions. Its large genome (~6.5 Gb) is highly enriched in transposable elements, with retrotransposons representing a major fraction of genomic content [27,28]. This TE-rich genomic landscape suggests that locusts may have evolved mechanisms to exploit TE activity for adaptive functions. In addition, locusts are major agricultural pests, making the elucidation of their antiviral defense mechanisms of both fundamental and applied significance [29].

Here, we investigate the role of a Gypsy-family retrotransposon (*LmGypsy*) during infection with *Acrididae reovirus* (ARV). We combine transcriptomic profiling, functional perturbation, and small RNA analysis to examine how *LmGypsy* activity influences antiviral responses. Our results show that *LmGypsy* activation is required for efficient antiviral defense and is associated with both vDNA production and the induction of cGAS-like signaling. These findings provide new evidence that retrotransposons can function as integrators of multiple immune pathways, and support the idea that TE-mediated antiviral mechanisms are conserved across diverse insect lineages.

## 2. Materials and Methods

### 2.1. Locusts Rearing

*Locusta migratoria* eggs in this study were obtained from Hebei Province Locust Research Base (China). To eliminate potential ARV contamination, eggs were surface-sterilized with a formaldehyde-hydrochloric acid mixture (3% *v*/*v* formaldehyde, 1% HCl) for 5 min, followed by three rinses in sterile phosphate-buffered saline (PBS, Servicebio, G4202, Wuhan, China). Eggs were incubated under aseptic conditions at 30 ± 1 °C and 70 ± 5% relative humidity (RH) until hatching. Newly emerged nymphs were maintained in controlled-environment chambers under standardized conditions (30 ± 1 °C, 70 ± 5% RH, 16:8 h light: dark photoperiod) and provided with ad libitum access to freshly germinated wheat seedlings (*Triticum aestivum*). Developmental stages were synchronized by selecting newly molted 3rd-instar nymphs for all experiments.

### 2.2. ARV Virions Preparation

ARV virions were purified from its natural host, *Dasyhippus barbipes*, as previously described [30]. Briefly, forty individuals were homogenized in ice-cold PBS (pH 7.4, Sigma-Aldrich, P5493, St. Louis, MO, USA) using a sterile tissue grinder (Jingxin, Shanghai, China). The homogenate was centrifuged at 1500× *g* for 10 min at 4 °C to remove tissue debris. Clarified supernatant was layered onto a pre-chilled discontinuous sucrose gradient (30%, 40%, 50%, 60% *w*/*v* in PBS) and ultracentrifuged at 240,000× *g* for 4 h at 4 °C in a SW 41 Ti rotor (Beckman Coulter, Brea, CA, USA). The visible virus-containing bands at the sucrose interface were aspirated with spinal needles (BD Biosciences, Franklin Lakes, NJ, USA). The collected viral fraction was diluted 1:10 (*v*/*v*) in sterile PBS, and further pelleted by ultracentrifugation at 240,000× *g* for 2 h at 4 °C. The resulting pellet was resuspended in PBS and aliquoted for storage at −80 °C. ARV virions were verified through quantitative real-time PCR (qPCR) with the following pairs of primers: forward 5′-CTCCATCTCTCCGAAGTAACTC-3′; reverse 5′-ACTGATCGATTGCGAGGTTC-3′ before proceeding with downstream applications. Virion morphology was additionally examined by transmission electron microscopy (TEM).

### 2.3. Transmission Electron Microscopy (TEM)

For negative staining, 20 µL of the purified virus suspension was adsorbed onto carbon-coated copper grids for 5 min. The grid was then washed with distilled water and stained with 3% phosphotungstic acid (pH 7.0, Macklin, P769541, Shanghai, China) for 1 min. Samples were observed under Hitachi H-7500 transmission electron microscope (Hitachi High-Technologies, Tokyo, Japan). Representative micrographs were captured to visualize the morphology and purity of the viral particles.

### 2.4. ARV Infection and Survival Assays

Newly molted third-instar nymphs were intracoelomically injected with 9 × 10^6^ virions in 1 μL PBS using a Nanoject III microinjector (Drummond Scientific, Broomall, PA, USA). Control nymphs received equivalent volumes of sterile PBS (pH 7.4, Sigma-Aldrich, P5493, St. Louis, MO, USA). To monitor viral load dynamics, ARV genomic RNA was quantified in whole-body homogenates at 24 h intervals by qPCR. Primers targeting the RNA-dependent RNA polymerase (*RdRP*) were used: forward 5′-CTCCATCTCTCCGAAGTAACTC-3′ and reverse 5′-ACTGATCGATTGCGAGGTTC-3′. Survival assays were conducted over 27 days post-infection (dpi), with mortality recorded daily. All experiments included five biological replicates (*n* = 15 per replicate).

### 2.5. RNA Isolation and Transcriptional Profiling

Total RNA was extracted from whole nymphs using TRIzol™ Reagent (Thermo Fisher Scientific, 15596026CN, Waltham, MA, USA) and treated with DNase I (Thermo Fisher Scientific, EN0521, Waltham, MA, USA) to eliminate genomic DNA. RNA integrity was verified using Bioanalyzer 2100 system (Agilent Technologies, Santa Clara, CA, USA). Strand-specific mRNA libraries were constructed from poly(A)-selected RNA (1 μg per sample) using the TruSeq Stranded Total RNA Kit (Illumina, RS-122-2203, San Diego, CA, USA) and sequenced on a NovaSeq 6000 platform (2 × 150 bp paired-end reads; Novogene, Beijing, China).

Raw reads were processed using Cutadapt v1.9 to remove adapter sequences, poly-A/G tails, reads containing >5% unknown nucleotides (N), and low-quality reads (with >20% of bases having a Q-value ≤ 20). Clean reads were evaluated with FastQC v0.11.9 for Q20, Q30 scores, and GC content. The filtered reads were aligned to the P50 reference genome using HISAT2 v2.2.1, allowing up to two mismatches and multiple alignments per read. Gene expression was quantified with StringTie v2.1.6 and Ballgown, and normalized as fragments per kilobase of transcript per million mapped reads (FPKM). Differential expression analysis was performed using DESeq2 v2_1.52.0 and edgeR v4.10.0. Genes with a false discovery rate (FDR) < 0.05 and an absolute fold change ≥2 were considered differentially expressed genes (DEGs). Functional enrichment analysis of DEGs was conducted using Gene Ontology (GO) and Kyoto Encyclopedia of Genes and Genomes (KEGG) pathway analyses based on the hypergeometric test. GO terms or KEGG pathways with a *p*-value < 0.05 were considered significantly enriched.

### 2.6. vDNA Detection and Small RNA Sequencing

To assess retrotransposon-mediated reverse transcription of ARV RNA, total DNA was isolated from infected nymphs (*n* = 15 per time point) using the TIANamp Genomic DNA Kit (Tiangen, DP304-02, Beijing, China). To eliminate viral RNA contamination, the extracted DNA were treated with RNase A and RNase III to degrade single-stranded and double-stranded RNA, respectively. Briefly, 10 µg of total DNA was incubated with RNase A (10 mg/mL, Solarbio, R1030, Beijing, China) and RNase III (100 U/µg, MedChemExpress, HY-KE7056, Monmouth Junction, NJ, USA) at 37 °C for 30 min, followed by purification using phenol–chloroform extraction and ethanol precipitation. ARV-derived vDNA was quantified by qPCR using multiple primer pairs that span the entire ARV genome (Supplementary Table S1). To validate the removal of RNA contamination, RNase-treated DNA sample was subjected to reverse transcription–quantitative PCR (RT-qPCR) with or without reverse transcriptase (RT) using the same primer sets. Purified ARV RNA was used as a positive control to verify the efficiency of the reverse transcription reaction. The relative quantification (2^−ΔΔCt^) method was used to compare the amplification signals between the +RT and −RT conditions. The absence of a statistically significant difference (*p* > 0.05, paired *t*-test) between the two conditions confirmed that the qPCR signals were derived from vDNA rather than residual RNA. For the positive control (ARV RNA), a significant increase in signal was observed only in the +RT condition, confirming the competency of the RT reaction.

To determine whether vDNA induces virus-derived small interfering RNAs (vsiRNAs), 3rd-instar nymphs were inoculated with 1 μg RNase-treated DNA purified from either ARV-infected or PBS-infected locusts. At 3 dpi, total RNA was extracted from 15 pooled nymphs per condition and subjected to small RNA sequencing. Small RNAs of 18–30 nt were enriched using the mirVana miRNA Isolation Kit (ThermoFisher, AM1561), and sequenced on an Illumina HiSeq 2500 platform. Raw reads were processed using Cutadapt v1.9 and FastQC v0.11.9 to remove adapter sequences, low-quality reads (Phred score < 20), and low-complexity sequences. High-quality reads were retained and aligned to the ARV genome using Bowtie2 v2.5.4. Mapped reads were extracted with SAMtools v1.20, and their size distribution and strand specificity were analyzed using the viRome V0.10 R package.

### 2.7. Phylogenetic and Functional Analysis of cGAS-like Receptors and STING in L. migratoria

In mammals and *Drosophila*, *cGAS* is a cytosolic nucleic acid sensor that triggers innate immune activation via its conserved downstream effector *STING* [31,32]. To elucidate the antiviral immune function of the cGAS-STING pathway in locusts, homologs of *cGAS* (designated *LmcGAS*) and *STING* (*LmSTING*) were first identified in *L. migratoria*. Reference sequences of *cGAS* and *STING* from representative vertebrates and invertebrates were retrieved from the NCBI database. These sequences were used as queries for BLASTp v2.17.0 searches (E-value < 1 × 10^−5^) against three published *L. migratoria* transcriptome datasets (GJPL00000000.1, GETS00000000.1, GBDZ00000000.1). Candidate sequences were validated by PCR amplification and Sanger sequencing. Functional domains were annotated using InterProScan v5.75-106.0. Phylogenetic trees were constructed using IQ-TREE v2.4.0 with 1000 ultrafast bootstrap replicates. For the *cGAS* phylogenetic tree, the structurally related mammalian oligoadenylate synthetase 1 (*OAS1*) and male abnormal 21 (*Mab21*) were also aligned and constructed the phylogenetic tree.

To assess transcriptional responses to viral infection, newly molted 3rd-instar nymphs were injected with ARV (9 × 10^6^ virions in 1 μL PBS). Whole-body samples were collected at 1 and 3 dpi. Total RNA was extracted using TRIzol™ Reagent (ThermoFisher, 15596026CN), and gene expression levels of *LmcGAS* and *LmSTING* were quantified by qPCR. qPCR was performed on a LightCycler^®^ 96 system using TB Green™ Premix Ex Taq II (Takara, CN830S, Shiga, Japan), with each sample run in triplicate technical replicates. Relative expression was normalized to housekeeping gene *β-actin* using the 2^−ΔΔCt^ method. Five independent biological replicates were performed, each with at least 15 nymphs per group.

### 2.8. RNA Interference (RNAi) Assays

Double-stranded RNAs (dsRNAs) were synthesized in vitro using the T7 RiboMAX Express RNAi System (Promega, P1700, Madison, WI, USA) according to the manufacturer’s instructions. Primers containing T7 promoter sequences (Supplementary Table S1) were used to amplify template DNA from *L. migratoria* cDNA. For knockdown efficiency validation, newly molted 3rd-instar nymphs were injected with 1 μg of the respective dsRNA. Total RNA was extracted at 1 and 3 dpi, and gene silencing efficiency was assessed by qPCR. Each experiment included five independent biological replicates with 15 nymphs per group.

### 2.9. Functional Analysis of Antiviral Genes by RNA Interference

To evaluate the antiviral roles of candidate genes, RNAi was performed in newly molted 3rd-instar nymphs. For each gene, dsRNA was synthesized as described above, and nymphs were injected with 1 μg of the respective dsRNA. A dsRNA targeting *GFP* (dsGFP) was used as a negative control. At 24 h post-injection (hpi), locusts were challenged with 9 × 10^6^ ARV virions. Viral loads were quantified from whole-body samples at 72 hpi by qPCR.

For *LmGypsy*, three independent dsRNAs targeting non-overlapping regions were designed to minimize potential off-target effects. For the cGAS-STING pathway, dsRNAs targeting *LmcGASs* or *LmSTING* were used. To determine the contribution of major antiviral pathways, key immune genes including *Dicer2* and *Argonaute2* (*Ago2*, RNAi pathway), *Myd88* (Toll pathway), and *Domeless* (JAK/STAT pathway) were individually knocked down. For each experiment, five independent biological replicates were used, each comprising 15 nymphs per group.

### 2.10. Tissue-Specific Expression Analysis of LmcGAS Genes Following ARV Infection

To investigate the tissue-specific expression patterns of *LmcGAS* genes during antiviral responses, newly molted third-instar nymphs were injected with ARV as described above. At 3 dpi, multiple tissues, including head (HD), epidermis (EP), fat body (FB), midgut (MG), ovary (OV), testis (TS), malpighian tubule (MT), and hemolymph (HL), were dissected under sterile conditions.

Total RNA was extracted from each tissue using TRIzol reagent (ThermoFisher, 15596026CN) according to the manufacturer’s instructions. For each biological replicate, tissues from five individuals were pooled to minimize inter-individual variation and to ensure sufficient RNA yield. Five independent biological replicates were analyzed for each tissue. Following DNase I treatment, first-strand cDNA was synthesized using a reverse transcription kit (Takara, RR092S, Shiga, Japan) according to the manufacturer’s protocol.

Relative transcript levels of *LmcGAS1–4* were quantified by qPCR using gene-specific primers (Supplementary Table S1). Expression values were normalized to *β-actin* as the internal reference gene and calculated using the 2^−ΔΔCt^ method. For each gene, the tissue displaying the lowest expression level was used as the calibrator for relative quantification. Statistical analyses were performed using one-way ANOVA followed by Tukey’s multiple-comparison test. Differences were considered statistically significant at *p* < 0.05.

### 2.11. AZT Treatment

To assess the role of *LmGypsy* in antiviral immunity, newly molted third-instar nymphs were starved for 12 h and then fed with fresh wheat leaf segments (0.5 cm × 0.5 cm) coated with 20 μL of 93 mM AZT (3′-azido-3′-deoxythymidine, Solarbio, IZ02909, Beijing, China) or sterile PBS as a control. AZT is a specific reverse transcriptase inhibitor and has no known inhibitory effect on the *RdRP* of reoviruses. Nymphs were allowed to feed on treated leaves for 24 h and then randomly assigned to four groups: (1) PBS control (PBS pretreatment + PBS inoculation), (2) AZT treatment (AZT pretreatment + PBS inoculation), (3) ARV infection (PBS pretreatment + ARV inoculation), and (4) AZT + ARV (AZT pretreatment + ARV inoculation). At 3 dpi, total RNA was extracted from whole nymphs using TRIzol™ Reagent (ThermoFisher, 15596026CN) according to the manufacturer’s instructions. cDNA was synthesized for quantitative PCR analysis. LmGypsy transcription levels and ARV viral loads were quantified by qPCR under identical conditions. Relative expression levels were calculated using the 2^−ΔΔCt^ method. A total of five independent biological replicates were carried out, each containing 15 nymphs per group.

### 2.12. Statistical Analyses

All statistical analyses in this study were performed using GraphPad Prism v9.5. Differences between groups were evaluated using one-way ANOVA or Student’s *t*-test as appropriate. Data are presented as mean ± standard error of the mean (SEM). Statistical details, including sample size, are provided in the figure legends. A *p* value ≤ 0.05 was considered statistically significant.

### 2.13. Ethics Statement

This study utilized the migratory locust (*L. migratoria*), an invertebrate insect species. The experimental procedures employed in this study, such as viral infection, behavioral observation, and tissue dissection, are standard practices in insect pathology and virology research. None of these procedures are considered to cause significant pain, distress, or harm beyond that inherent in routine laboratory rearing. Locusts used in this study were obtained from Hebei Province Locust Research Base (Hebei, China).

## 3. Results

### 3.1. ARV-Triggered Activation of a Gypsy Retrotransposon Contributes to Antiviral Immunity in L. migratoria

**Figure 1:**
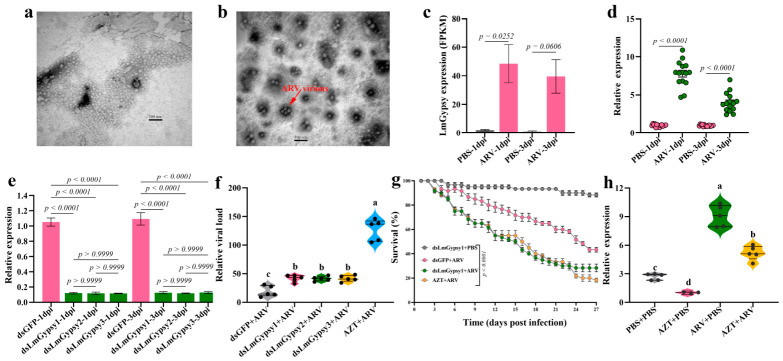
**ARV infection-induced activation of *LmGypsy* retrotransposon is required for antiviral immunity and host survival in *L. migratoria*.** (**a**) Transmission electron microscopy (TEM) image of the control group. No obvious virions were observed. (**b**) TEM of purified ARV virions. (**c**) RNA-seq analysis of *LmGypsy* transcript levels in ARV-infected or PBS mock-treated nymphs at 1 and 3 dpi. Transcript levels are shown as fragments per kilobase of transcript per million mapped reads (FPKM). (**d**) qPCR validation of *LmGypsy* upregulation upon ARV infection. (**e**) RNAi-mediated knockdown efficiency of *LmGypsy*. (**f**) LmGypsy knockdown or AZT treatment increases viral load at 3 dpi. (**g**) LmGypsy knockdown or AZT treatment reduces survival after ARV infection (27-day monitoring). (**h**) AZT treatment suppresses ARV-induced *LmGypsy* expression. For qPCR analyses, expression levels were normalized to *β-actin*. Viral loads were quantified by qPCR. Each experiment included five biological replicates (15 nymphs per replicate). Data are shown as mean ± standard error of the mean (SEM). Statistical significance was determined using unpaired two-tailed Student’s *t*-test for pairwise comparisons (**c**–**e**,**g**) or one-way ANOVA with Tukey’s post hoc test for multiple comparisons (**f**,**h**). Different letters indicate significant differences among groups determined by one-way ANOVA followed by Tukey’s multiple-comparison test (*p* < 0.05).

The ARV strain used in this study was isolated from its natural host. Its identity was previously confirmed by TEM morphology and complete genome sequencing [31]. TEM imaging showed the typical morphology of reovirus particles (Figure 1a,b). Based on this well-established virus–host system, we next sought to comprehensively characterize the genes and immune pathways involved in antiviral responses in *L. migratoria*. Following ARV infection in 3rd-instar nymphs, transcriptome sequencing was performed at 1 and 3 dpi. Remarkably, a retrotransposon annotated as the Gypsy element (*LmGypsy*) was significantly upregulated as early as 1 dpi (*p* = 0.0252; Figure 1c). This observation was further validated by qPCR analyses, which revealed a dramatic increase in *LmGypsy* expression following ARV infection (*p* < 0.0001; Figure 1d). This rapid induction of *LmGypsy* suggests its potential role in antiviral defenses, aligning with emerging evidence that the TEs reactivation can contribute to antiviral immunity in virus-infected hosts [32,33].

To further investigate the role of *LmGypsy* during ARV infection, we performed loss-of-function assays using RNAi. Three independent dsRNAs (dsLmGypsy1, 2, 3) targeting distinct regions of *LmGypsy* were designed. All three dsRNAs efficiently reduced *LmGypsy* mRNA levels (>80% vs. dsGFP, Figure 1e), with no statistically significant difference among them (Figure 1e). Importantly, no detectable developmental defects or abnormal phenotypes were observed in dsRNA-treated nymphs, indicating negligible toxicity. We subsequently assessed the impact of *LmGypsy* knockdown on viral replication using these independent dsRNAs. Compared with the dsGFP-treated, *LmGypsy*-knockdown nymphs exhibited significantly higher viral loads at 3 dpi (*p* < 0.05; Figure 1f), indicating a marked impairment of antiviral immunity. Notably, consistent effects on viral replication were observed across all three dsLmGypsy treatments (Figure 1e), confirming that the phenotype was specifically attributable to *LmGypsy* knockdown. Based on these results, dsLmGypsy1 was selected for subsequent functional assays. To evaluate the physiological relevance of *LmGypsy*-mediated antiviral defense, we monitored the survival of ARV-challenged nymphs over a 27-day period. *LmGypsy* knockdown significantly reduced survival rates (*p* < 0.0001; Figure 1g), with survival decreasing from 43.33% in the control group to 28.33% in the knockdown group. These findings collectively indicate that *LmGypsy* contributes to antiviral immunity and enhances host survival during ARV infection.

Concurrently, we examined the effects of AZT treatment, a reverse transcriptase inhibitor, on *LmGypsy* expression and antiviral responses. qPCR analysis confirmed that ARV infection induced a significant upregulation of *LmGypsy* expression (ARV + PBS vs. PBS control; *p* < 0.05; Figure 1h). AZT treatment alone significantly reduced basal *LmGypsy* expression (AZT + PBS vs. PBS control; *p* < 0.05), and importantly, AZT pretreatment almost completely abrogated the virus-induced upregulation of *LmGypsy* (AZT + ARV vs. ARV + PBS; *p* < 0.05; Figure 1h). These results demonstrate that AZT effectively suppresses both basal and infection-induced transcription of *LmGypsy*. We next investigated whether AZT-mediated suppression of *LmGypsy* affects antiviral immunity. In line with our RNAi results, AZT treatment led to a significant increase in ARV viral loads (*p* < 0.05; Figure 1f) and a concomitant reduction in the survival of ARV-infected nymphs (*p* < 0.0001; Figure 1g). Together, these findings support a role for ARV-induced *LmGypsy* activation in locust antiviral immunity. They also raise the possibility that TE-mediated immune pathways could be explored for future disease resistance strategies.

### 3.2. L. migratoria Produces ARV-Derived DNA via LmGypsy Activity

**Figure 2:**
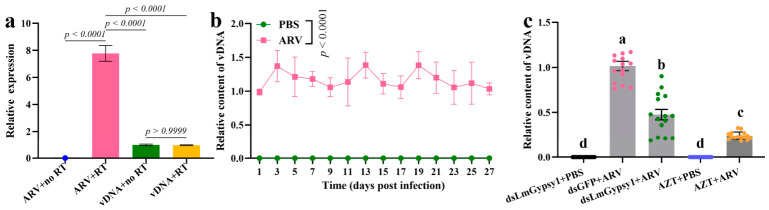
**ARV infection induces *LmGypsy*-dependent vDNA production in locusts.** (**a**) RT-qPCR validation of RNA removal efficiency. (**b**) Detection of ARV-derived vDNA in locust nymphs at various time points post-infection. (**c**) LmGypsy knockdown or AZT treatment significantly reduces vDNA levels at 3 dpi. vDNA was quantified by qPCR targeting the ARV RdRP gene. Bars represent mean ± SEM from five biological replicates (15 nymphs each). For each treatment, five nymphs were pooled per biological replicate, and three measurements were generated from each replicate, resulting in 15 total measurements per group. ND indicates values below detection limits. Statistical significance was assessed using unpaired two-tailed Student’s *t*-test (**a**,**b**) or one-way ANOVA with Tukey’s test (**c**). All qPCR reactions were performed in technical triplicates. Different letters (a–d) indicate significant differences among groups based on one-way ANOVA (*p* < 0.05).

Non-retroviral RNA viruses can be reverse-transcribed into vDNA by host retrotransposons or endogenous retroviruses, a mechanism documented across mammals, plants, and multiple insect orders including Diptera and Lepidoptera [34,35,36]. However, whether this mechanism extends to other arthropods, particularly Orthopteran insects such as *L. migratoria*, remains unclear. To investigate the presence of ARV-specific vDNA, genomic DNA was extracted from ARV-infected nymphs. To exclude viral RNA contamination, DNA samples were treated with RNase prior to amplification. The purified DNA was then amplified using multiple primer pairs spanning the entire ARV genome. To validate the efficiency of RNA removal, the RNase-treated DNA samples were subjected to RT-qPCR with or without reverse transcriptase using the same primer sets. No statistically significant difference was observed between the +RT and −RT conditions (*p* > 0.05, Figure 2a), confirming that the qPCR signals originated from vDNA rather than residual viral RNA. A positive control using purified ARV RNA showed a significant signal only in the +RT condition, verifying the competency of the reverse transcription reaction (Figure 2a). Following RNase treatment, ARV-derived DNA was amplified using multiple primer pairs spanning the entire ARV genome. Amplifiable products were consistently obtained only with primers targeting the complete RdRP coding sequence, as confirmed by Sanger sequencing, whereas no amplification was detected with primers targeting other genomic regions.

Using a pair of primers specifically designed for qPCR, we dynamically profiled vDNA synthesis kinetics. vDNA was detectable as early as 24 hpi and persisted throughout the infection period in locusts (Figure 2b). These data confirm that ARV infection triggers rapid and sustained production of vDNA in *L. migratoria*. To further assess whether ARV-derived vDNA could access the germline, we examined vDNA accumulation in reproductive tissues and offspring derived from ARV-infected locusts. No detectable vDNA signal was observed in either ovaries or testes following ARV infection. Consistently, vDNA was also undetectable in eggs produced by infected adults after mating. These findings suggest that ARV-derived vDNA is unlikely to be transmitted through the germline in *L. migratoria* under our experimental conditions. Given that retrotransposon-mediated reverse transcriptase activity has been implicated in vDNA formation in *Drosophila*, mosquitoes, and silkworms [22,23,37], we therefore hypothesized that the ARV-induced *LmGypsy* retrotransposon mediates vDNA synthesis in locusts. Indeed, RNAi-mediated knockdown of *LmGypsy* resulted in a significant reduction in vDNA production (*p* < 0.05; Figure 2c). Furthermore, treatment with AZT further diminished vDNA synthesis (*p* < 0.05; Figure 2c), supporting a role of reverse transcription in this process. Collectively, these results indicate that *LmGypsy* contributes to the production of ARV-derived vDNA in *L. migratoria*, likely through a reverse transcription–dependent mechanism.

### 3.3. ARV-Derived vDNA Generates vsiRNAs That Activate Antiviral RNAi Pathway

**Figure 3:**
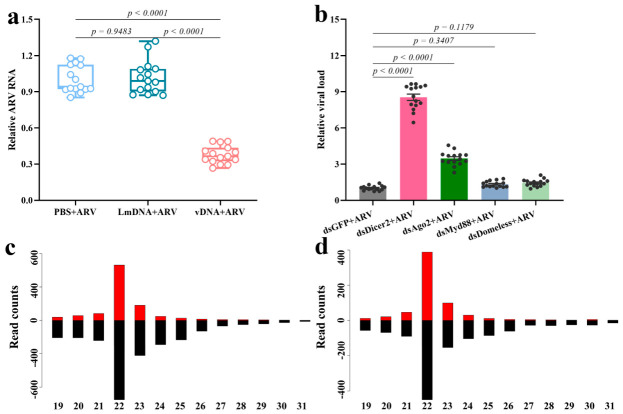
**ARV-derived vDNA suppresses viral replication by activating the antiviral RNAi pathway in *L. migratoria*.** (**a**) Antiviral effect of vDNA pre-inoculation on ARV replication. Nymphs were pre-inoculated with vDNA (from ARV-infected locusts), LmDNA (from uninfected locusts) or PBS control 24 h before ARV challenge; viral loads were quantified by qPCR at 72 hpi. (**b**) Effects of immune pathway knockdown on ARV replication. At 24 h post-dsRNA injection, nymphs were infected with ARV. Viral loads were quantified at 72 hpi by qPCR, and expressed as relative fold change compared to the dsGFP control group. (**c**) Size distribution of vsiRNAs in vDNA-inoculated nymphs, determined by small RNA sequencing at 3 dpi. (**d**) Size distribution of vsiRNAs in ARV-infected nymphs at 3 dpi. Data are presented as mean ± SEM from five biological replicates, each containing 15 nymphs. Each dot represents one measurement derived from pooled samples of five nymphs. Three measurements were obtained for each biological replicate, giving 15 measurements per treatment group. Statistical significance in (**a**,**b**) was determined using unpaired two-tailed Student’s *t*-test for pairwise comparisons. For (**c**,**d**), data are representative of two independent small RNA sequencing experiments, each performed with pooled samples from 15 nymphs. Red bars represent small RNAs mapped to the positive strand, whereas black bars represent small RNAs mapped to the negative strand.

vDNAs have been reported to execute an antiviral function in insects [22,23,37]. To evaluate whether ARV-derived vDNA could protect locust from viral infection, 3rd-instar nymphs were pre-inoculated with total DNA isolated from ARV-infected (vDNA) or uninfected locusts (LmDNA) 24 h prior to ARV challenge. At 72 hpi, vDNA-pretreated nymphs exhibited significantly lower viral titer than LmDNA-treated controls (*p* < 0.0001; Figure 3a), confirming that ARV-derived vDNA mediates antiviral protection in locusts. Previous studies have proposed that vDNA enhances RNAi-mediated immunity by promoting production of antiviral vsiRNAs [37,38]. To investigate the underlying mechanism in *L. migratoria*, we individually knocked down key components of major antiviral pathways [39]. Knockdown of *Dicer-2*, or *Ago2* led to a significant ARV accumulation at 72 hpi (*p* < 0.0001; Figure 3b). whereas knockdown of *Myd88* (*p* = 0.3407) or *Domeless* (*p* = 0.1179) had no significant effect on viral loads (Figure 3b). These data formally establish that RNAi is the primary antiviral pathway in this context. We next performed small RNA sequencing to directly test whether ARV-derived vDNA drives the production of antiviral vsiRNAs. Nymphs were inoculated with vDNA or LmDNA, and small RNA profiles were generated at 3 dpi. Strikingly, vDNA delivery induced the specific production of ARV-derived siRNAs with two canonical hallmarks of *Dicer-2*-dependent antiviral vsiRNAs: (i) perfect complementarity to the ARV RdRP sequence; and (ii) a bimodal size distribution (19–31 nt) with a predominant peak at 22 nt, consistent with *Dicer-2* processing of dsRNA precursors (Figure 3c) [40]. In contrast, no virus-specific siRNAs were detected in LmDNA-inoculated control nymphs. Furthermore, the size distribution and sequence features of vDNA-induced siRNAs fully recapitulated the vsiRNAs profile observed in nymphs with active ARV infection (Figure 3c,d). This finding provides direct evidence that ARV-derived vDNA acts as a template for vsiRNA biogenesis, thereby activating the canonical siRNA pathway to mediate antiviral immunity in *L. migratoria.*.

### 3.4. LmGypsy-Dependent Activation of cGAS-like Signaling Contributes to Antiviral Immunity in L. migratoria

**Figure 4:**
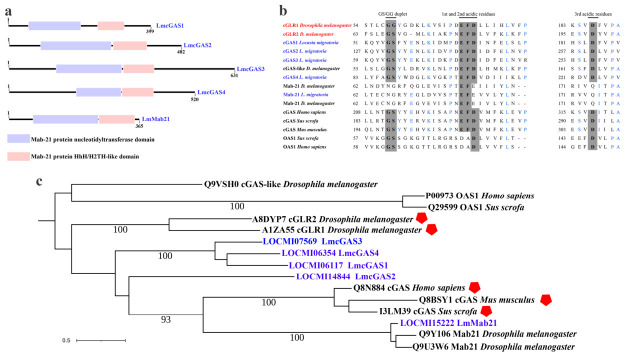
**Identification and structural characterization of cGAS-like candidates in *L. migratoria*.** (**a**) Domain organization of LmcGAS1–4 and LmMab21. Conserved domains were predicted using InterProScan and are shown as colored boxes: blue, NTase domain; orange, Mab21 domain. Protein lengths (amino acids) are indicated on the right. The numbers shown correspond to the sequence start and end coordinates. (**b**) Multiple sequence alignment of LmcGASs and LmMab21 with representative cGAS-like receptors from *Drosophila* (cGLRs and Mab21) and mammals (cGAS and OAS1). Conserved catalytic motifs, including the GS/GG dinucleotide and metal ion-coordinating acidic residues, are highlighted above the alignment. Identical and similar residues are shaded according to conservation scores. (**c**) Maximum likelihood phylogenetic tree of cGAS, OAS1, and Mab21 family proteins. The tree was constructed using IQ-TREE v3.1.2 with 1000 ultrafast bootstrap replicates. Bootstrap support values are indicated at branch nodes. Proteins with experimentally validated antiviral functions are marked with red pentagons. The *L. migratoria* candidates identified in this study (LmcGAS1–4 and LmMab21) are highlighted in blue.

Exogenous pathogens can induce the derepression of host transposable elements, leading to the production of immunostimulatory nucleic acid species capable of activating innate immune pathways [41,42]. In mammals and *Drosophila*, cGAS-like receptors function as conserved cytosolic sensors involved in antiviral defense [43,44]. We therefore investigated whether ARV infection induces cGAS-like signaling in *L. migratoria,* and whether this process is associated with *LmGypsy* activity. A genome-wide homology search identified four putative cGAS-like genes in the *L. migratoria* genome (LmcGAS1–4, Figure 4a). Domain architecture analysis revealed that all four *LmcGAS* candidates harbor the two conserved structural domains and a canonical active site required for cyclic dinucleotide (CDN) synthase activity, a defining feature of functional cGAS orthologs (Figure 4a,b). We additionally identified a Mab21-like protein (LmMab21) that clustered within the cGAS-Mab21 subfamily but lacked the conserved catalytic residues required for CDN synthesis (Figure 4a,b). Phylogenetic analysis further showed that LmcGASs grouped closely with *Drosophila* cGLRs (Figure 4c), a family of pattern-recognition receptors known tomediate antiviral innate immunity in flies [45]. To investigate the downstream signaling machinery associated with these receptors, we identified and characterized the locust homolog of *STING*, a core downstream effector of cGAS signaling. This analysis identified a single *STING* homolog in *L. migratoria* (designated *LmSting*).

**Figure 5:**
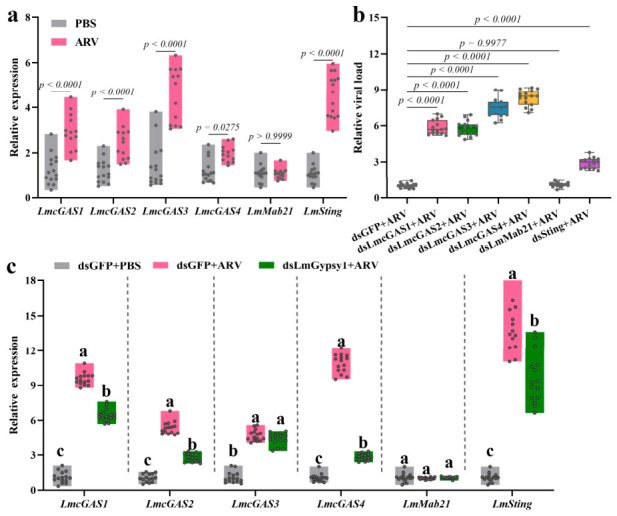
***LmcGAS*-mediated antiviral immunity requires LmGypsy activation and signals through the *Sting* pathway**. (**a**) Transcriptional responses of LmcGAS1–4, LmMab21, and LmSting following ARV infection. (**b**) ARV viral loads in nymphs after RNAi knockdown of *LmcGAS1–4*, *LmMab21*, or *LmSting*. (**c**) Expression of *LmcGAS1–4*, *LmMab21*, and *LmSting* in ARV-infected nymphs under *LmGypsy* knockdown, or corresponding controls. Different letters above bars denote significant differences at *p* < 0.05 (one-way ANOVA). Viral loads and gene expression were quantified by qPCR at 3 dpi and normalized to *β-actin*. Data are shown as mean ± SEM from five biological replicates. Each replicate consisted of pooled samples from five nymphs and was represented by three independent measurements, resulting in 15 data points per treatment group. Statistical significance was determined using unpaired two-tailed Student’s *t*-test (**a**,**b**) or one-way ANOVA with Tukey’s test (**c**). The dots represent individual measurements from all biological replicates.

To determine whether these candidates participate in antiviral immunity, we first examined their transcriptional responses following ARV infection. ARV challenge significantly induced the expression of *LmcGAS1–4* and *LmSting*, whereas *LmMab21* expression remained largely unchanged (Figure 5a). We next evaluated their antiviral functions using RNAi-mediated knockdown assays. Silencing of *LmcGAS1–4* or *LmSting* significantly increased ARV accumulation relative to the dsGFP control, whereas knockdown of *LmMab21* had no detectable effect on viral replication (Figure 5b). These findings indicate that *LmcGASs* and *LmSting* contribute to antiviral defense in *L. migratoria*. Given the virus-induced activation of both *LmGypsy* and the cGAS-like pathway, we next explored a potential functional connection between these responses. RNAi-mediated depletion of *LmGypsy* significantly reduced the infection-induced expression of *LmcGAS1, LmcGAS2, LmcGAS4*, and *LmSting* (Figure 5c). In contrast, *LmcGAS3* and *LmMab21* expression showed little or no response to *LmGypsy* knockdown. Together, these results suggest that *LmGypsy* activity contributes to the activation of a subset of cGAS-like signaling components during ARV infection.

**Figure 6:**
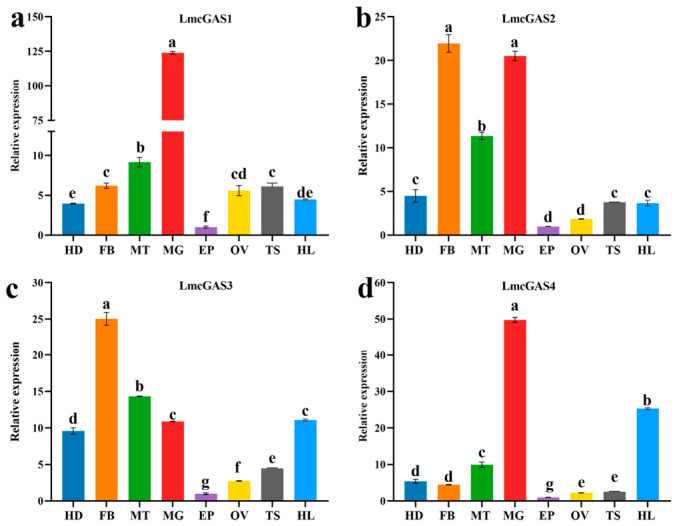
**Tissue-specific expression profiles of LmcGAS1–4 in ARV-infected locusts at 3 dpi.** (**a**–**d**) Relative expression levels of LmcGAS1, LmcGAS2, LmcGAS3, and LmcGAS4 in different tissues at 3 dpi following ARV infection. Transcript levels were quantified by qPCR and normalized to *β-actin*. For each gene, the tissue exhibiting the lowest expression level was used as the calibrator for relative quantification. HD, head; EP, epidermis; FB, fat body; MG, midgut; OV, ovary; TS, testis; MT, malpighian tubule; HL, hemolymph. Each biological replicate consisted of pooled tissues from five individuals, and five independent biological replicates were analyzed. Data are presented as mean ± SEM. Different letters above bars indicate statistically significant differences among tissues (one-way ANOVA followed by Tukey’s multiple-comparison test, *p* < 0.05). Different letters (a–g) above the bars indicate significant differences among groups (one-way ANOVA, *p* < 0.05).

To further explore the potential functional divergence among the four *LmcGAS* receptors, we examined their tissue-specific expression profiles at 3 dpi following ARV infection. Distinct expression patterns were observed across tissues. *LmcGAS1* was predominantly expressed in the midgut, whereas *LmcGAS2* showed highest expression in the fat body and midgut. In contrast, *LmcGAS3* was most strongly enriched in the fat body, with relatively high expression also detected in the malpighian tubule. *LmcGAS4* exhibited dominant expression in the midgut and comparatively elevated levels in the hemolymph (Figure 6). These distinct spatial expression patterns support the idea that individual *LmcGAS* receptors may perform partially specialized immune functions in different tissues during antiviral responses.

## 4. Discussion

**Figure 7:**
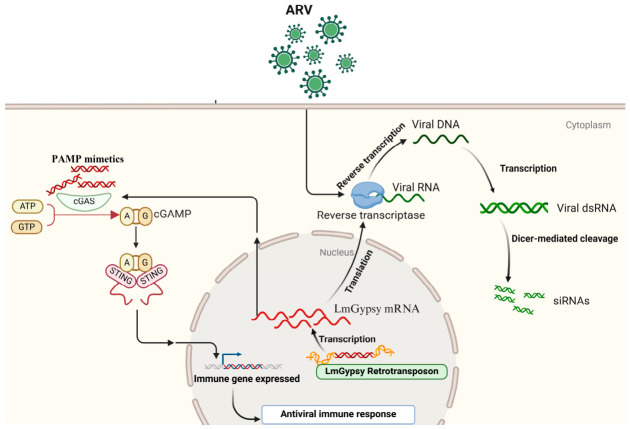
**Dual antiviral pathways mediated by LmGypsy through RNAi amplification and cGAS-like signaling in *L. migratoria*.** ARV infection triggers selective derepression of the LmGypsy retrotransposon, which contributes to two complementary antiviral mechanisms: (1) LmGypsy activity promotes the production of viral-derived DNA (vDNA), which contributes to vsiRNA biogenesis and enhances RNAi-mediated antiviral defense. (2) LmGypsy activity is required for LmcGAS-LmSting-mediated antiviral immunity. One possibility is that ARV-induced *LmGypsy* derepression produces nucleic acid ligands that activate LmcGAS signaling, though the molecular nature of these ligands remains to be determined.

In this study, we uncover a sophisticated antiviral mechanism in the *L. migratoria*, wherein the Gypsy retrotransposon *LmGypsy* acts as a central hub that coordinates the RNAi pathway and cGAS-like receptor signaling (Figure 7). This dual-layer defense establishes *LmGypsy* as a critical immune sentinel. Moreover, it provides compelling evidence for the evolutionary conservation of TE-mediated antiviral strategies across metazoans, bridging findings from mammalian systems and insects [14,40,41].

A key finding of our work is that ARV infection triggers the selective derepression of *LmGypsy*, and that its activity is essential for the accumulation of ARV-derived vDNA. Similar mechanisms have been reported in *Drosophila*, mosquitoes, and silkworms [22,23,37,38], where retrotransposon-derived reverse transcriptases promote the formation of antiviral vDNA intermediates. Consistently, AZT treatment reduced both LmGypsy expression and vDNA accumulation, partially recapitulating the effects of LmGypsy knockdown. Notably, ARV-derived vDNA persisted from 24 hpi until host death, indicating that these molecules are not merely transient replication intermediates. Such persistence may contribute to sustained antiviral activity or prolonged immune priming within infected individuals. In *Drosophila melanogaster*, virus-derived DNA has been reported to participate in forms of immune memory and, in some cases, has been associated with heritable antiviral responses [46,47]. However, in our study, we did not detect ARV-derived vDNA in either ovaries, testes, or eggs produced by infected adults, arguing against efficient germline transmission in *L. migratoria* under the conditions tested. These findings suggest that the persistence of vDNA in locusts is more likely linked to somatic antiviral defense rather than transgenerational inheritance. Nevertheless, although our data support a functional link between *LmGypsy* activity and vDNA accumulation, they do not formally distinguish between direct and indirect mechanisms underlying this relationship. In particular, the concurrent reduction in *LmGypsy* transcription and vDNA levels following AZT treatment points to a potentially more complex regulatory relationship between retrotransposon activity and vDNA biogenesis. Moreover, because long terminal repeat retrotransposons (LTR) frequently harbor enhancer and promoter activities capable of modulating neighboring genes [48,49,50], silencing LmGypsy may indirectly affect host factors involved in antiviral defense or nucleic acid metabolism. Future genetic dissection of the *LmGypsy* locus and its RT domain will therefore be required to resolve these possibilities.

Beyond fueling RNAi, we reveal that the reactivation of *LmGypsy* engages a parallel, complementary pathway through cGAS-like receptors (*LmcGASs*). Our functional data suggest that *LmGypsy* activity contributes to the activation of *LmcGAS1/2/4* and *LmSting* during ARV infection. Supporting this, the infection-induced upregulation of these factors is abrogated by both *LmGypsy* knockdown and AZT-mediated RT inhibition. This positions *LmGypsy*-derived products as critical upstream ligands for *LmcGASs*, mirroring the mammalian cGAS-STING pathway in sensing endogenous retroelements (e.g., *LINE-1*) to potentiate antiviral immunity [44]. Nevertheless, direct evidence linking *LmGypsy* activity to pathogen-associated molecular pattern (PAMP) generation and *LmcGAS* binding remains incomplete. We did not directly visualize immunostimulatory nucleic acids upon AZT treatment, nor did we provide biochemical proof of physical interaction between *LmcGASs* and *LmGypsy*-derived ligands. Future studies using anti-dsRNA/RNA:DNA hybrid antibodies, in vitro binding assays, and structural analyses will be required to determine whether AZT reduces PAMP levels and whether *LmcGASs* directly sense retrotransposon-derived nucleic acids. This will clarify whether insect *cGLRs* have evolved specialized TE-sensing capabilities or act as general sensors activated by TE-derepression milieus.

From an evolutionary perspective, our findings underscore a remarkable convergence between insect and mammalian antiviral strategies. In both systems, controlled TE derepression during viral infection generates immunostimulatory nucleic acids. These molecules are subsequently recognized by conserved nucleic acid-sensing pathways, including cGAS/cGLR signaling, thereby amplifying antiviral defense [14,44,51]. This suggests that TE-immune system crosstalk represents an ancient and fundamental layer of antiviral immunity. However, the patterns of TE activation appear to differ substantially among organisms. In mammals, viral infection often induces broad derepression of multiple retroelement families, including *LINE-1* and *SINEs* (short interspersed nuclear elements), which collectively contribute to cytosolic nucleic acid accumulation and activation of the cGAS-STING pathway [15,52,53]. Such broad TE activation may reflect the greater immunological complexity of vertebrates and the extensive reliance on interferon-mediated antiviral signaling. By contrast, TE activation in insects appears more selective and context dependent. Previous studies in *Drosophila* [38], mosquitoes, and silkworms have shown that only a subset of retrotransposons contribute to antiviral vDNA production or RNAi amplification [23,24,37,38]. Consistent with this pattern, our data indicate that ARV infection preferentially induces a specific LTR retrotransposon, *LmGypsy*, rather than causing widespread derepression of multiple TE families. This selective activation may provide important evolutionary advantages by balancing antiviral immune amplification with preservation of genomic stability. Broad TE activation could increase the risk of insertional mutagenesis, genome instability, transcriptional dysregulation, and excessive immune activation, all of which may compromise host fitness [48,54]. In contrast, restricting antiviral activity to a limited subset of retrotransposons may allow the host to exploit the immunostimulatory properties of TE-derived nucleic acids [54,55]. At the same time, such selective activation could minimize the genomic instability associated with uncontrolled transposition. Such fine-tuned regulation may reflect an evolutionarily constrained interaction between the host and specific retrotransposons [56]. Recurrent viral infections may have promoted the evolutionary retention of TE families with immunostimulatory potential [57,58]. Concurrently, hosts likely evolved regulatory mechanisms to restrict excessive TE mobilization and preserve genomic stability [57,58]. In this context, *LmGypsy* may represent a retrotransposon with increased immune responsiveness that has become functionally integrated into antiviral defense pathways in *L. migratoria*.

The double-edged nature of TE activation is a central theme in this field [13,15]. While our study highlights its benefits in antiviral defense, the mechanisms that restrict *LmGypsy* activation to prevent genomic instability remain unknown. In mammals, epigenetic silencers like Tripartite motif-containing 28 (TRIM28) can be transiently inactivated during viral infection, releasing retrotransposons for immune benefit [59,60]. A similar mechanism may exist in locusts, where ARV infection might subvert host chromatin modifiers to derepress *LmGypsy*. Deciphering the upstream regulatory logic that governs *LmGypsy* expression will be a critical next step, as it could reveal how the host balances the beneficial and detrimental consequences of TE activity.

Finally, our work challenges the traditional view of insect antiviral immunity as being primarily RNAi-centric. Instead, it presents a more integrated model where RNAi and nucleic acid-sensing pathways converge on a common TE hub. Functional divergence among the four LmcGASs, particularly the LmGypsy-independent behavior of LmcGAS3, suggests receptor specialization. Supporting this idea, the four receptors displayed distinct tissue-specific expression patterns following ARV infection. For example, *LmcGAS3* was predominantly expressed in the fat body and malpighian tubule, whereas LmcGAS1 and *LmcGAS4* were strongly enriched in the midgut. These differences may reflect tissue-adapted sensing strategies or recognition of distinct nucleic acid ligands during antiviral responses. Although the ligand preferences of individual *LmcGAS* receptors remain unresolved, our findings provide initial evidence that the cGAS-like signaling system in locusts is functionally diversified rather than redundant. In conclusion, our study identifies a critical role for the *LmGypsy* retrotransposon in orchestrating antiviral immunity in a major agricultural pest. Furthermore, it establishes a powerful model for dissecting the conserved principles of TE-host coevolution in the context of host–pathogen conflicts.

## Data Availability

The raw reads of RNA-seq and sRNA data generated in this study are available at the NCBI SRA database with BioProject accessions PRJNA1206438 and PRJNA1206808. The nucleotide sequences of *LmGypsy*, *LmcGASs*, and *LmMab21* are available in Supplementary Table S2.

## Supplementary Materials

The following supporting information can be downloaded at: https://www.mdpi.com/article/10.3390/insects17060539/s1.
